# Cloning and molecular characterization of an ovine ABCD4 cDNA sequence, recombinant protein expression, and polyclonal antibody generation in Ujimqin sheep

**DOI:** 10.3389/fvets.2026.1869570

**Published:** 2026-10-28

**Authors:** Yue Zhang, Jiajia Zhang, Jianglin Zhao, Lin Jiang, Qihuan Zhao, Jiahui Wu, Ruizhen Jian, Guoqiang Yuan, Size Zhao, Guifang Cao, Yong Zhang, Fuxiang Bao, Jun Liu

**Affiliations:** 1 College of Veterinary Medicine, Northwest A&F University, Yangling, Shaanxi, China; 2 College of Veterinary Medicine, Inner Mongolia Agricultural University, Hohhot, China; 3 Inner Mongolia Academy of Agricultural & Animal Husbandry Sciences, Hohhot, China; 4 Animal Embryo and Developmental Engineering Key Laboratory of Higher Education, Institutions of Inner Mongolia Autonomous Region, Hohhot, China

**Keywords:** ABCD4, cDNA cloning, polyclonal antibody, prokaryotic expression, RACE, Ujimqin sheep

## Abstract

**Introduction:**

ATP-binding cassette subfamily D member 4 (ABCD4) is an ATP-binding cassette transporter involved in intracellular cobalamin trafficking and has also been identified as a candidate gene associated with vertebral-number variation in livestock. However, molecular information and experimental resources for ovine *ABCD4* remain limited. This study aimed to characterize a RACE-derived *ABCD4* cDNA sequence from Ujimqin sheep, analyze its sequence and predicted molecular features, establish a recombinant protein expression system, and generate a polyclonal antibody resource for subsequent studies.

**Methods:**

Total RNA isolated from Ujimqin sheep liver was subjected to rapid amplification of cDNA ends (RACE), followed by sequence analysis, phylogenetic comparison, physicochemical characterization, recombinant expression in Escherichia coli, protein purification, and antibody production.

**Results:**

A 1,874-bp *ABCD4* cDNA sequence was obtained, containing a predicted 1,209-bp open reading frame encoding a 402-amino-acid polypeptide. Sequence and bioinformatic analyses showed that the RACE-derived sequence retained conserved features of the ABC transporter family, although its predicted protein length was shorter than that of canonical mammalian ABCD4 proteins. For recombinant antigen production, the *ABCD4* expression construct was introduced into E. coli BL21 (DE3), and higher recombinant protein expression was observed after induction with 0.5 mM IPTG at 37 °C for 4 h. The recombinant protein was predominantly recovered from the insoluble fraction and purified by Ni-NTA affinity chromatography. Purified recombinant protein was then used to immunize New Zealand White rabbits solely for polyclonal antiserum production, and the resulting antisera showed strong immunoreactivity against the recombinant antigen.

**Discussion:**

Overall, this study provides a RACE-derived ovine *ABCD4* cDNA sequence together with recombinant protein and antibody resources that may support future investigations of *ABCD4* expression, localization, and biological function in sheep.

## 1 Introduction

ATP-binding cassette subfamily D member 4 (ABCD4) belongs to the ATP-binding cassette (ABC) transporter superfamily, whose members utilize ATP hydrolysis to drive the transmembrane transport of diverse substrates (1–4). Canonical full-size ABC transporters generally contain two transmembrane domains (TMDs) and two nucleotide-binding domains (NBDs), whereas mammalian ABCD proteins are half-transporters, with each monomer containing one TMD and one NBD and functioning as a dimer (4–6). The mammalian ABCD subfamily comprises ABCD1–4. ABCD1–3 are predominantly localized to peroxisomal membranes and participate mainly in the transport of fatty-acyl substrates into peroxisomes (5, 7–9), whereas ABCD4 differs substantially in both subcellular localization and transport direction. ABCD4 is primarily localized to lysosomal membranes, where it mediates the export of cobalamin from the lysosomal lumen to the cytoplasm, and its lysosomal targeting depends on LMBD1-mediated trafficking from the endoplasmic reticulum to lysosomes (5, 7, 10, 11). Structurally, a full-length ABCD4 protomer contains a six-helix transmembrane domain (TM1–TM6) and a cytosolic nucleotide-binding domain containing highly conserved ABC transporter motifs involved in ATP binding and hydrolysis; two protomers associate to form the functional transporter containing two TMDs and two NBDs (6, 12). Functionally, circulating cobalamin bound to transcobalamin is internalized through CD320-mediated endocytosis and delivered to lysosomes, from which free cobalamin is exported by ABCD4 and subsequently converted into methylcobalamin and adenosylcobalamin, the active cofactors of methionine synthase and methylmalonyl-CoA mutase, respectively (10, 13, 14). Accordingly, loss-of-function mutations in ABCD4 impair lysosomal cobalamin export and cause methylmalonic acidemia with homocystinuria, cblJ type, an autosomal recessive disorder of intracellular vitamin B12 metabolism that may present with hypotonia, failure to thrive, bone marrow suppression, anemia, and lethargy (13, 15–21). Disruption of vitamin B12 metabolism may also influence broader metabolic and signaling processes, including the Wnt pathway (16).

In recent years, genome-wide association studies (GWAS) and population genetics analyses have made substantial progress in identifying genes related to important economic traits in livestock, revealing numerous potential candidate genes. Among these, *ABCD4* has been demonstrated as a candidate gene associated with certain economic traits, including variations in vertebral number. For instance, GWAS in Beijing Black pigs and Ujimqin sheep identified *ABCD4* as a key gene influencing rib number (22–24) and vertebral number, respectively (25). Despite its potential genetic importance, the molecular features, transcript structure, and physicochemical properties of ovine *ABCD4* remain largely uncharacterized.

Accordingly, this study aimed to characterize a RACE-derived *ABCD4* cDNA sequence from Ujimqin sheep, analyze its sequence and predicted molecular features, establish a recombinant protein expression system, and generate polyclonal antisera for subsequent studies.

## 2 Materials and methods

### 2.1 Ethical statement

All experimental procedures in this study were approved by the Animal Care and Use Committee of Inner Mongolia Agricultural University, Inner Mongolia Autonomous Region, China (Permit No. 2020008).

### 2.2 Experimental materials

One-month-old male Ujimqin lambs were obtained from the Ujimqin Stud Farm in Inner Mongolia. Liver tissues were collected from two one-month-old male Ujimqin lambs and used as biological sources for RNA extraction and *ABCD4* cDNA cloning. Liver was selected as the RNA source because publicly available expression information indicated relatively high *ABCD4* expression in this tissue, thereby facilitating recovery of the target transcript. The use of male lambs was not based on a known sex-specific role of *ABCD4*; animals of the same age and sex were used to maintain consistency in the biological source material. The two lambs were used for molecular cloning rather than as a population-representative sample, and the study was not designed to assess population- or sex-related differences in *ABCD4* expression. Liver tissues were aseptically collected, immediately snap-frozen in liquid nitrogen, and stored at −80 °C until further use. The remaining tissues and slaughter waste were subjected to harmless treatment procedures for safe disposal. In addition, two healthy female New Zealand White rabbits at 4 months of age with an average body weight of approximately 2.1 kg were used solely for polyclonal antibody production against the recombinant ABCD4 antigen, and all experimental animals were raised and handled following standard animal welfare and ethical protocols.

### 2.3 Experimental methods

#### 2.3.1 RACE amplification of ovine ABCD4 cDNA

Total RNA was extracted from liver tissue using TRIzol reagent (Sangon Biotech, Shanghai, China). RNA purity and concentration were assessed using a NanoDrop 2000 spectrophotometer (Thermo Fisher Scientific, USA), and RNA integrity was evaluated by agarose gel electrophoresis. Gene-specific primers for 5' RACE (ABCD4-NRT1, ABCD4-NRT2, ABCD4-NR1, and ABCD4-NR2) and 3' RACE (ABCD4-NF1 and ABCD4-NF2), together with the corresponding adaptor primers, were synthesized by Sangon Biotech (Shanghai, China) (Table 1).

**Table 1:** Oligonucleotide primers used in the experiments.

Primer name	Sequence (5^′^ → 3^′^)
5′adaptor	GCTGTCAACGATACGCTACGTAACGGCATGACAGTGGGIIGGGIIGGGIIG
3′adaptor	GCTGTCAACGATACGCTACGTAACGGCATGACAGTGTTTTTTTTTTTTTTTTTT
5.3′outer	GCTGTCAACGATACGCTACGTAAC
5.3′inner	GCTACGTAACGGCATGACAGTG
ABCD4-NF1	AGGACAAAGGGTCTGGACCAG
ABCD4-NF2	TTTGCCCGGCTCTTCTACCT
ABCD4-NRT1	CCCGTGTTGCCTGTGATG
ABCD4-NRT2	CTTCAGACTCAGGTCCTTGATTAAC
ABCD4-NR1	CTGAAGCAGTTGATGAGATACAAGC
ABCD4-NR2	CCGTAGACGCCACTGAAAATG
Nde I-ABCD4	CATATGCATCACCACCACCACCATTCAGGAGT
Hind III-ABCD4	AAGCTTTCATTACTCGACCTTAATACGGGTT

For 3′ RACE, first-strand cDNA was synthesized using the 3′ RACE System Kit (Sangon Biotech, Shanghai, China) with the 3′ adaptor primer as the reverse-transcription primer. Nested PCR was performed in two rounds, each in a total reaction volume of 20 μL. The first-round reaction contained 0.5 μL of ABCD4-NF1 (10 μM), 0.5 μL of 5.3′ outer (10 μM), 0.5 μL of cDNA template, 13.5 μL of Taq SuperMix, and RNase-free water to 20 μL. For the second-round PCR, diluted first-round PCR product was used as the template, with ABCD4-NF2 and 5.3′ inner as the primer pair. The cycling conditions for both rounds are summarized in Table 2.

**Table 2:** PCR cycling conditions for 5′ and 3′ RACE amplification.

Step	Temperature (°C)	Time	Number of cycles
Initial denaturation	94	60 s	1
Denaturation	94	30 s	10
Touchdown annealing	70–61^*^	30 s	10
Extension	72	2 min	10
Denaturation	94	30 s	25
Annealing	60	30 s	25
Extension	72	2 min	25
Final extension	72	8 min	1

^*^The annealing temperature was decreased by 1 °C per cycle during the first 10 touchdown cycles.

For 5′ RACE, first-strand cDNA was synthesized using the 5′ RACE System Kit (Sangon Biotech, Shanghai, China) with ABCD4-NRT1 as the reverse-transcription primer. Briefly, 0.5–2 μg of total RNA, 1 μL of ABCD4-NRT1, and RNase-free water to a final volume of 10 μL were mixed, incubated at 65 °C for 5 min, and immediately chilled on ice for 2 min. Subsequently, 10 μL of Reverse Transcriptase Mix (RNase H–) was added, followed by incubation at 50 °C for 30 min and enzyme inactivation at 85 °C for 1 min. The resulting cDNA was treated with RNase H, purified, and subjected to terminal deoxynucleotidyl transferase (TdT)-mediated poly(dC) tailing according to the manufacturer's instructions. The poly(dC)-tailed cDNA was then used for two rounds of nested PCR in a total reaction volume of 20 μL. The first-round PCR used ABCD4-NR1 and 5'adaptor as the primer pair. A diluted first-round PCR product was subsequently used as the template for the second-round PCR with ABCD4-NR2 and 5.3′ outer. The cycling conditions for both rounds are summarized in Table 2.

#### 2.3.2 Cloning and sequencing of RACE products

RACE amplification products were separated by agarose gel electrophoresis and purified using the SanPrep Column DNA Gel Extraction Kit (Sangon Biotech, Shanghai, China). The purified products were cloned into the pMD18-T vector and transformed into competent cells. Recombinant colonies were screened by colony PCR using the vector-specific M13+(-47) and M13–(-48) primers. Positive clones were subjected to Sanger sequencing by Sangon Biotech (Shanghai, China). The resulting sequences were subsequently assembled and used for sequence comparison and bioinformatic analyses.

#### 2.3.3 ABCD4 gene sequence analysis

The RACE-derived *ABCD4* cDNA sequences were assembled and analyzed using Contig Express 9.1. The deduced amino acid sequence of Ujimqin sheep ABCD4 was aligned with ABCD4 protein sequences retrieved from the NCBI database, including sheep (*Ovis aries*, XP_060274318.1), goat (*Capra hircus*, XP_005686131.1), cattle (*Bos taurus*, XP_059746924.1), human (*Homo sapiens*, XP_054232484.1), mouse (*Mus musculus*, XP_006515676.1), pig (*Sus scrofa*, XP_001929231.2), horse (*Equus caballus*, XP_001490430.2), donkey (*Equus asinus*, XP_014691270.2), and wild camel (*Camelus ferus*, XP_032338252.1). Multiple sequence alignment was performed using DNAMAN software. A phylogenetic tree was constructed in MEGA 11.0 using the neighbor-joining method with 1,000 bootstrap replicates.

Conserved domains were identified using the NCBI Conserved Domain Database (CDD) and the CD-Search tool, and the results were visualized using TBtools. The ExPASy server (http://www.expasy.org) was used to analyze the physicochemical properties, hydrophobicity, and secondary structure of the protein. Physicochemical parameters were calculated using ProtParam (https://web.expasy.org/protparam/), hydrophobicity was analyzed using ProtScale, and secondary structure was predicted using SOPMA (Self-Optimized Prediction Method with Alignment). The tertiary structure of the ABCD4 protein was predicted by homology modeling using the SWISS-MODEL server (http://swissmodel.expasy.org/interactive), and the optimal model was selected for subsequent structural analysis.

#### 2.3.4 Prokaryotic expression of ovine ABCD4 protein

Gene-specific primers, Nde I-ABCD4 and Hind III-ABCD4 (Table 1), were designed based on the coding sequence of *ABCD4* and incorporated Nde I and Hind III restriction sites, respectively. PCR amplification was performed using the verified cDNA as template and 2 × Phanta Max Master Mix, set as: initial denaturation at 95 °C for 3 min; 30 cycles of denaturation at 95 °C for 15 s, annealing at 61 °C for 15 s, and extension at 72 °C for 72 s; a final extension at 72 °C for 5 min. The purified PCR product and the pET-30a(+) vector were digested with Nde I and Hind III. The digested products were purified and ligated using T4 DNA ligase overnight at 16 °C. The ligation mixture was transformed into E. coli DH5α competent cells and spread on LB agar plates containing kanamycin (50 μg/mL). After overnight incubation at 37 °C, single colonies were picked and screened by colony PCR and sequencing to identify positive recombinant clones. The sequence-verified plasmid was then transformed into *E. coli* BL21 (DE3) cells and designated pET-30a-ABCD4.

Three individual colonies of *E. coli* BL21 (DE3) carrying pET-30a-ABCD4 were inoculated into 4 mL of LB medium supplemented with kanamycin (50 μg/mL) and grown at 37 °C with shaking at 200 rpm until the OD_600_ reached 0.6. Two of the cultures were induced with IPTG at a final concentration of 0.5 mM, followed by incubation at 15 °C for 16 h or at 37 °C for 4 h. The third culture was maintained as an uninduced negative control. Following induction, the cells were collected by centrifugation at 12,000 rpm for 10 min, washed three times with PBS, and resuspended in the same buffer. Cell disruption was performed by ultrasonication on ice at 300 W using cycles of 2 s on and 3 s off for 1.5 h, with PMSF added to a final concentration of 1 mM every 30 min. The lysate was centrifuged at 10,000 × g for 10 min at 4 °C to separate the soluble fraction from the pellet. The pellet was resuspended in binding buffer containing 8 M urea and centrifuged again to collect the supernatant. Samples from each fraction, including the supernatant and pellet, were mixed with 5 × protein loading buffer, boiled for 10 min, and analyzed by SDS-PAGE at 150 V for 1 h 45 min. The gels were stained with SolarFast Protein Staining Buffer (Solarbio, Beijing, China) for 10 min, washed 2–3 times with distilled water, and photographed to assess the expression profile and identify the optimal induction conditions.

#### 2.3.5 Purification and identification of recombinant ovine ABCD4 protein

According to the SDS-PAGE analysis, the pellet fraction from the cell lysate of culture induced at 37 °C for 4 h, which showed the highest expression of the target protein, was chosen for purification by Ni-NTA gravity-flow column chromatography. The Ni-NTA column was equilibrated with binding buffer, after which the sample was loaded and incubated overnight at 4 °C to allow protein binding. The column was then washed three times with 10 mL of washing buffer containing 8 M urea, and the recombinant protein was subsequently eluted three times with 1 mL elution buffer. The eluted protein was renatured by gradient dialysis and stored at −80 °C until use. The samples were subsequently separated by SDS-PAGE and electrotransferred onto a methanol-activated PVDF membrane at 120 mA for 50 min. The membrane was blocked and then incubated with THE™ His Tag Antibody, mAb, Mouse (Genscript, Nanjing, China) at a dilution of 1:1,000 for 4 h. After washing six times with 1 × TBST for 5 min each, the membrane was incubated with ProteinFind^®^ Goat Anti-Mouse IgG (H+L), HRP Conjugate at a dilution of 1:5,000 for 4 h. Following another six washes with 1 × TBST for 5 min each, immunoreactive bands were visualized using an ECL chemiluminescence kit (Beyotime, Shanghai, China).

#### 2.3.6 Preparation and titer determination of polyclonal antibody against recombinant ovine ABCD4 protein

For the primary immunization, the purified recombinant protein was emulsified with an equal volume of Freund's complete adjuvant. For the second, third, and fourth booster immunizations, the antigen was emulsified with an equal volume of Freund's incomplete adjuvant. Two healthy female New Zealand White rabbits aged 4 months and weighing approximately 2.1 kg were used for polyclonal antibody production. The rabbits were immunized on Day 1 with 300 μg of recombinant protein emulsified in 750 μL of Freund's complete adjuvant. Booster immunizations were administered on Days 21, 35, and 49, each consisting of 200 μg of protein antigen emulsified in 750 μL of Freund's incomplete adjuvant. Blood samples (1 mL) were collected from the ear vein on Days 42 and 56 for ELISA-based antibody titer determination. After evaluation of the antibody response by ELISA, final bleeding was performed via the carotid artery on Day 57.

Antisera obtained after the third and fourth immunizations from both rabbits were subjected to indirect ELISA for antibody titer determination. The recombinant protein antigen was diluted in 0.05 mol/L carbonate buffer, pH 9.6, and used to coat 96-well plates at 0.2 μg per well in 100 μL overnight at 4 °C. The plates were washed three times with PBS containing 0.05% Tween-20 (PBST) for 3 min each and blocked with 150 μL of 5% skim milk for 90 min at 37 °C, followed by three additional washes with PBST. Rabbit sera were initially diluted 1:1,000 and then subjected to serial two-fold dilutions before incubation for 1 h at 37 °C. After washing, HRP-conjugated goat anti-rabbit IgG (H+L) (Sangon Biotech, Shanghai, China) diluted 1:8,000 was added and incubated for 45 min at 37 °C. The plates were washed five times with PBST, after which 100 μL of TMB substrate solution was added to each well and allowed to react for 15 min. The reaction was stopped with 100 μL of 2 mol/L H_2_SO_4_, and absorbance was measured at 450 nm using a microplate reader (Thermo Fisher, USA). A positive ELISA reaction was defined as an OD450 value ≥2.5 times that of the negative control. The endpoint titer was defined as the highest serum or antibody dilution that still met this criterion.

Western blot analysis was performed to evaluate the immunoreactivity of the anti-ABCD4 polyclonal antibody toward sheep liver proteins. The extracted proteins were separated by SDS-PAGE and transferred onto a PVDF membrane using a semi-dry transfer system (Bio-Rad, USA). After blocking, the membrane was incubated overnight at 4 °C with rabbit anti-ABCD4 polyclonal antibody serum at a dilution of 1:1,000. Following three washes with PBST, the membrane was incubated with HRP-conjugated goat anti-rabbit IgG (Sangon Biotech, Shanghai, China) for 1 h at room temperature. After additional washes with PBST, the specific protein bands were visualized using an appropriate detection reagent.

## 3 Results

### 3.1 RACE amplification and sequence characterization of ovine ABCD4 cDNA

Total RNA extracted from lamb liver was of high purity and integrity and was therefore suitable for subsequent RACE analysis (Figure 1A). The 5' RACE amplification yielded a single major product, whereas 3' RACE generated three products of different lengths (Figures 1B, C). Sequence comparison showed that products 1 and 2 shared the same predicted coding sequence, whereas product 3 diverged upstream of the predicted stop codon. When translated from the same internal AUG, products 1 and 2 were predicted to encode 402-amino-acid polypeptides, whereas product 3 was predicted to encode a 398-amino-acid polypeptide; the first approximately 380 amino acids were shared, but the C-terminal sequence of product 3 was altered. Because these RACE products were not independently validated as stable endogenous isoforms, they are referred to here as three 3' RACE products rather than confirmed transcript variants. The longest RACE-derived cDNA sequence obtained in this study was 1,874 bp. ORF analysis identified a 1,209-bp open reading frame extending from nucleotide 43 to nucleotide 1,251 and encoding a predicted 402-amino-acid polypeptide. Comparison with the annotated ovine ABCD4 RefSeq isoform X1 (XM_004010785.4; XP_004010834.2), which encodes a 606-amino-acid protein, showed that the predicted 402-amino-acid sequence corresponds to residues 205–606 of XP_004010834.2. Thus, the RACE-derived sequence lacks the N-terminal 204 amino acids present in the annotated X1 protein, indicating that the 5' coding region was not completely recovered. Additional annotated ovine ABCD4 models (XM_012182043.2/ XP_012037433.1, XM_012182044.2/ XP_012037434.1, and XM_012182046.2/ XP_012037436.1) encode proteins of 596, 589, and 516 amino acids, respectively, all longer than the 402-amino-acid polypeptide obtained here. Accordingly, the 1,874-bp sequence is described as a RACE-derived ovine *ABCD4* cDNA sequence rather than a full-length transcript; genomic sequence identity supports its origin from the *ABCD4* locus but does not establish transcript completeness.

**Figure 1:**
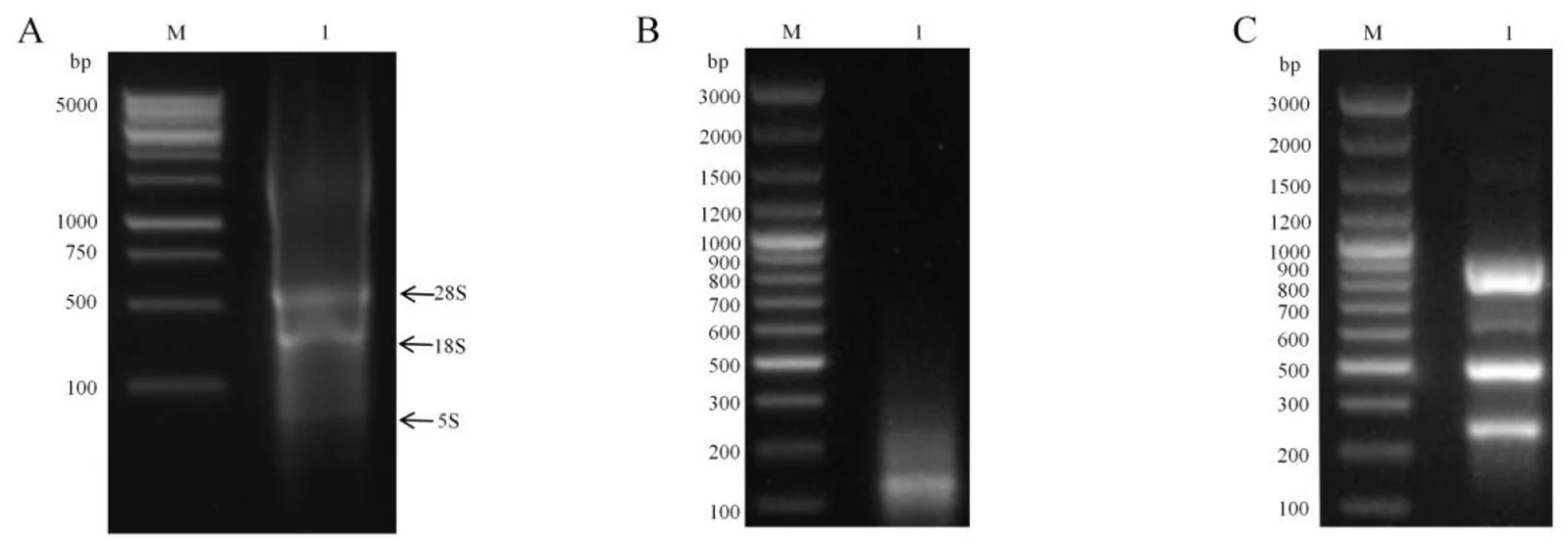
RACE amplification and sequence characterization of ovine *ABCD4* cDNA products. **(A)** Agarose gel electrophoresis of sheep liver total RNA to assess RNA integrity. M, DNA marker (bp); lane 1, total RNA. **(B, C)** Agarose gel electrophoresis of the5′ rapid amplification of cDNA ends (RACE) **(B)** and3′ RACE **(C)** amplification products.

### 3.2 Sequence characteristics and bioinformatics analysis of the Ujimqin sheep ABCD4 protein

Conserved-domain analysis using the NCBI Conserved Domain Database (CDD) identified an N-terminal ABC transporter membrane-associated region (ABC_membrane_2; Pfam06472) spanning residues 1–90 of the predicted 402-amino-acid polypeptide. A highly significant ABCD-subfamily ATP-binding cassette domain (ABCD_peroxisomal_ALDP; cd03223) was identified at residues 183–394 (E-value = 6.85 × 10^−78^). Consistently, a generic ABC transporter nucleotide-binding/ATPase domain (ABC_ATPase; cd00267) was detected within residues 196–383. These results support classification of the predicted protein as an ABCD-family transporter-related protein containing a membrane-associated N-terminal region and a conserved C-terminal nucleotide-binding domain. However, because the predicted polypeptide is shorter than canonical mammalian ABCD4 proteins, the present analysis does not establish the presence of the complete canonical ABCD4 transmembrane architecture.

Multiple sequence alignment of ABCD4 protein sequences from nine mammalian species showed that the amino acid sequence and functional domains of the Ujimqin sheep ABCD4 protein are highly conserved. A phylogenetic tree constructed using the neighbor-joining NJ method illustrated the evolutionary relationships among these species and showed that sheep ABCD4 clustered with those of goat and cattle (Figure 2A). Physicochemical analysis indicated that the ovine ABCD4 CDS predicted 402-amino-acid polypeptide with a predicted molecular weight of 45,283.04 Da. The predicted polypeptide contained five cysteine residues. The predicted molecular formula of the ovine ABCD4 protein was C_2036_H_3209_N_541_O_598_S^15^, and its theoretical isoelectric point (pI) was 5.43. Leucine (Leu) was the most abundant amino acid, with 57 residues (14.2%), followed by serine (Ser), with 33 residues (8.2%). The protein contained 41 positively charged and 50 negatively charged amino acid residues.

**Figure 2:**
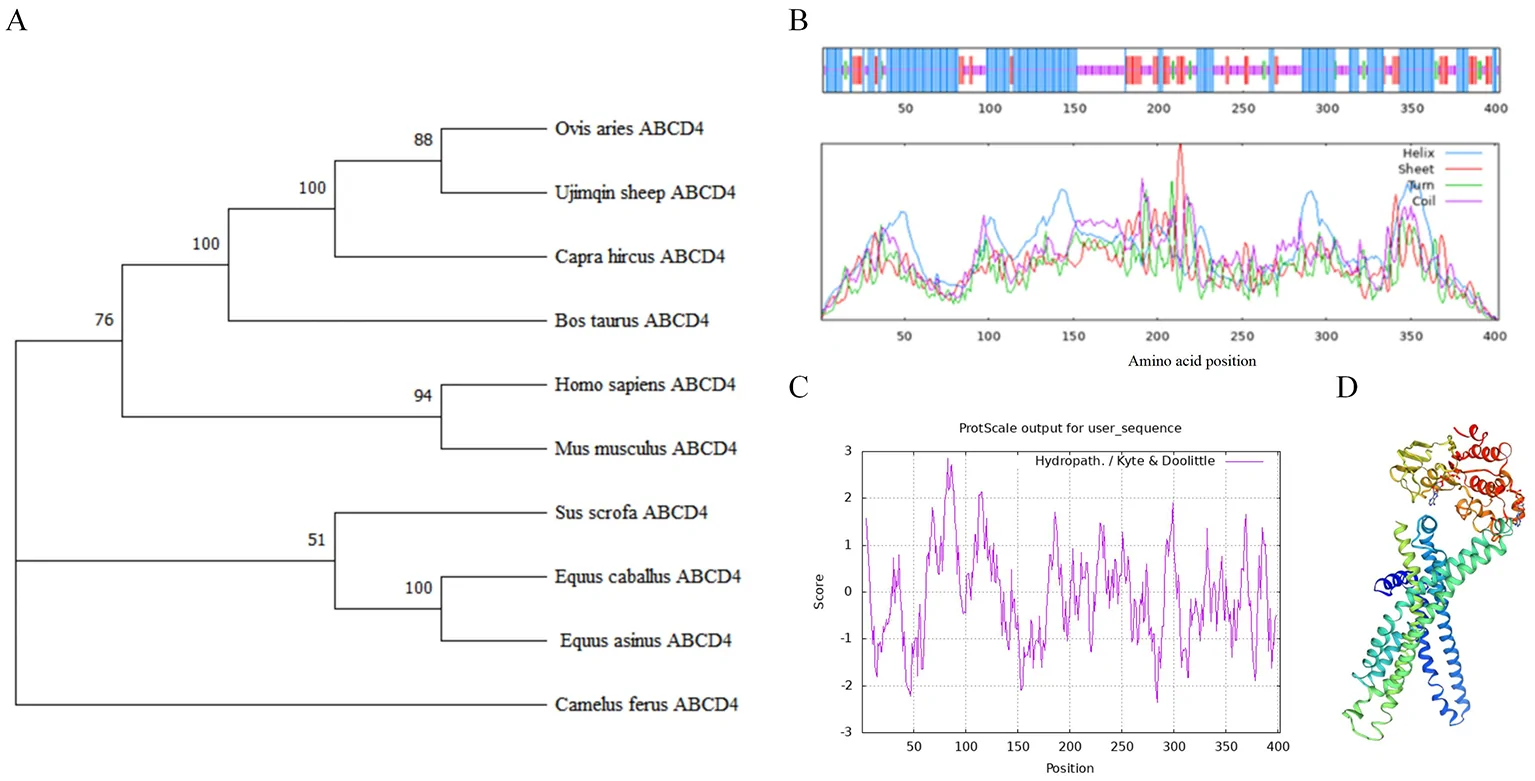
Phylogenetic analysis and structural characterization of the ovine ABCD4 protein. **(A)** Neighbor-joining NJ phylogenetic tree based on ABCD4 amino acid sequences from multiple species. **(B)** Predicted secondary structure of the ovine ABCD4 protein. **(C)** Hydrophobicity profile of the ovine ABCD4 protein, showing several strongly hydrophobic regions characteristic of transmembrane proteins. **(D)** Predicted tertiary structure model of the ovine ABCD4 protein.

SOPMA-based secondary structure prediction showed that the predicted ovine ABCD4 protein consisted predominantly of α-helices (49.25%) and random coils (31.09%), with smaller proportions of extended strands (15.42%) and β-turns (4.23%; Figure 2B). The average hydrophobicity was 0.092, indicating that the ovine ABCD4 protein is moderately hydrophobic. In addition, the instability index suggested that the protein is relatively stable (Figure 2C). SWISS-MODEL-based homology modeling selected a template sharing 88.56% sequence identity with the Ujimqin sheep ABCD4 protein and generated a three-dimensional structural model with a GMQE score of 0.85 and a QMEANDisCo score of 0.81 ± 0.05 (Figure 2D).

### 3.3 Expression, purification, and characterization of recombinant ABCD4 protein in *E. coli*

Agarose gel electrophoresis of the recombinant plasmid following double digestion showed two expected bands at approximately 7,000 bp and 1,000 bp, compared with the undigested plasmid, confirming the successful ligation of the target gene into the pET-30a(+) vector (Figures 3A, B). The recombinant plasmid was further confirmed by colony PCR and sequencing. The verified positive clone was subsequently transformed into E. coli BL21 (DE3) for expression analysis.

**Figure 3:**
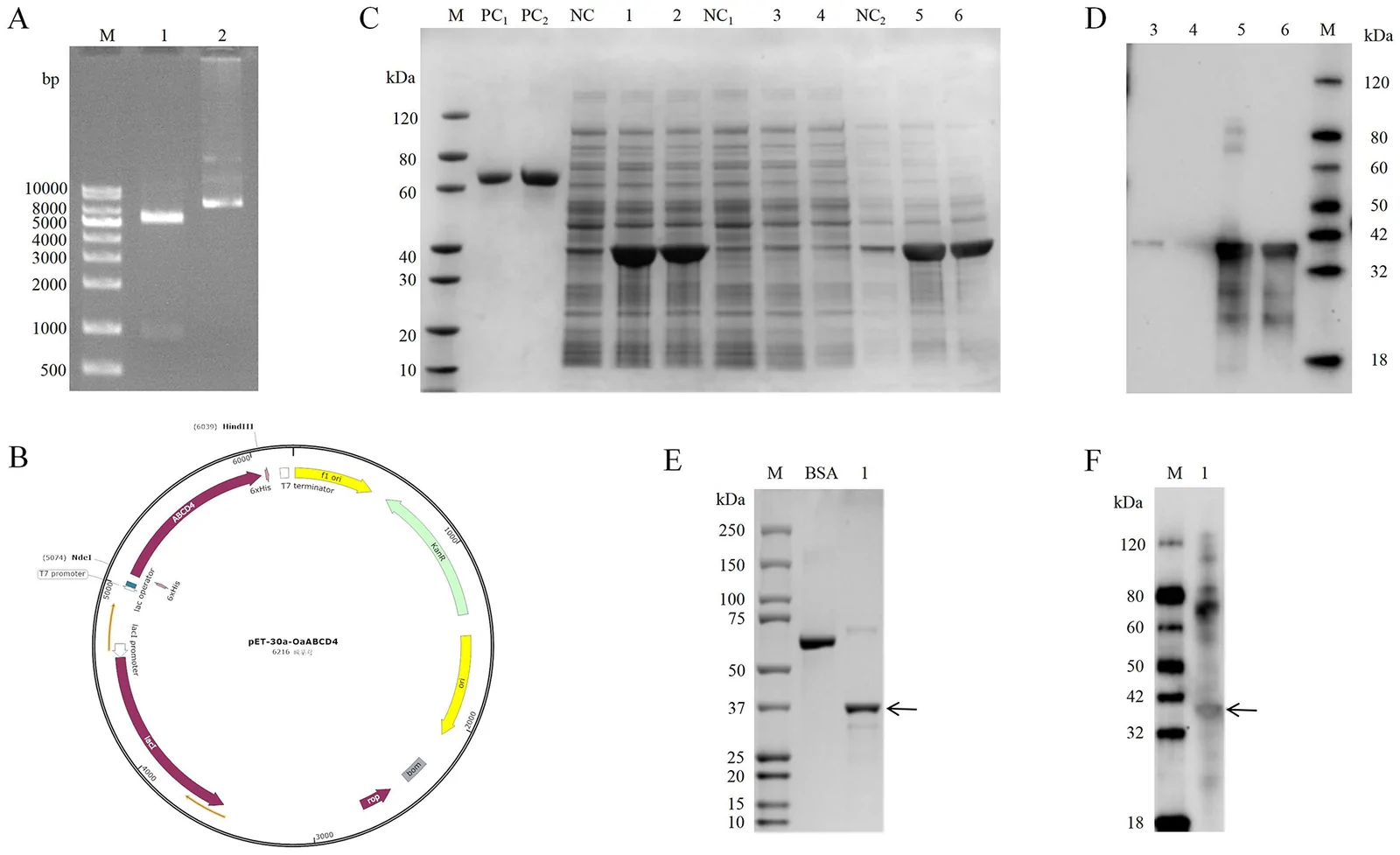
Construction of the recombinant pET-30a-ABCD4 plasmid and expression, purification, and identification of the recombinant ABCD4 protein. **(A)** Agarose gel electrophoretic of the double-digested recombinant expression vector. Lane 1: double-digested recombinant vector. Lane 2: undigested plasmid control. **(B)** Schematic diagram of the recombinant expression vector. **(C)** SDS-PAGE analysis of induced recombinant ABCD4 protein expression. M: protein marker kDa. PC1: BSA, 1 μg. PC2: BSA, 2 μg. NC: uninduced whole cells. Lane 1: whole cells induced at 15 °C for 16 h. Lane 2: whole cells induced at 37 °C for 4 h. NC1: supernatant from uninduced cell lysate. Lane 3: supernatant from cells induced at 15 °C for 16 h. Lane 4: supernatant from cells induced at 37 °C for 4 h. NC2: pellet from uninduced cell lysate. Lane 5: pellet from cells induced at 15 °C for 16 h. Lane 6: pellet from cells induced at 37 °C for 4 h. The results show enhanced expression of the target protein at 37 °C, predominantly in the inclusion body fraction. **(D)** Western blot analysis of the recombinant protein after sonication. M: protein marker kDa. Lanes 3–6 correspond to supernatant/pellet samples from different treatment groups, confirming the insoluble protein expression. **(E)** Ni-NTA affinity purification of the recombinant protein. M: protein marker kDa. BSA: 2 μg. Lane 1: purified recombinant ABCD4 protein. The arrow indicates a highly purified target protein band at approximately 37 kDa. **(F)** Western blot analysis of the purified recombinant protein using an anti-His antibody. The arrow indicates a major immunoreactive band with an apparent molecular mass of approximately 37 kDa.

Following cultivation, expression of the recombinant ABCD4 protein was induced with IPTG at 15 °C and 37 °C. SDS-PAGE analysis revealed a distinct target protein band of approximately 37 kDa in whole-cell lysates and insoluble fractions after IPTG induction. In this experiment, PC1 and PC2 represented 1 μg and 2 μg BSA as quantitative controls, NC referred to uninduced whole cells, NC1 and NC2 were the supernatant and pellet fractions of uninduced cell lysate, respectively; Lane 1 and Lane 2 were whole bacterial samples induced at 15 °C for 16 h and 37 °C for 4 h, Lane 3 and Lane 4 corresponded to the supernatant fractions under the two induction conditions, while Lane 5 and Lane 6 were the precipitated fractions accordingly. Specific target bands were clearly observed in induced whole cells and pellet samples, yet almost no visible target protein was detected in the supernatant. Collectively, the expression level of recombinant ABCD4 protein was higher at 37 °C, and the target protein was predominantly expressed in the insoluble pellet fraction in the form of inclusion bodies, (Figure 3C), which was further confirmed by Western blotting (Figure 3D). Protein expression levels were higher at 37 °C after 4 h of induction than at 15 °C after 16 h of induction. Most of the recombinant ABCD4 protein was detected in the inclusion body fraction, indicating that it was expressed predominantly in an insoluble form. Based on expression efficiency and band intensity, induction at 37 °C for 4 h was selected as the optimal condition. The resulting inclusion bodies were solubilized in 8 M urea, and the recombinant protein was purified by Ni-NTA affinity chromatography. SDS-PAGE revealed a highly purified His-tagged protein of approximately 37 kDa (Figure 3E). Western blot analysis using an anti-His monoclonal antibody detected a prominent immunoreactive band with an apparent molecular mass of approximately 37 kDa (Figure 3F). The calculated molecular mass of the N-terminally His-tagged recombinant protein was approximately 46 kDa, indicating a discrepancy between its calculated and apparent electrophoretic molecular masses.

### 3.4 Preparation and titer evaluation of anti-ABCD4 polyclonal antibody

Following four rounds of immunization, antibody responses were evaluated by indirect ELISA. A positive ELISA reaction was defined as an OD450 value ≥2.5 times that of the corresponding negative control, and the endpoint titer was defined as the highest dilution that still met this criterion. For the antisera collected after final bleeding, the endpoint titers were 1:64,000 for rabbit A and 1:256,000 for rabbit B (Figure 4A). Following antibody preparation, the endpoint titers were ≥1:512,000 for antibody A and ≥1:256,000 for antibody B (Figure 4B). These results demonstrated strong immunoreactivity of the antibody preparations against the recombinant antigen. The calculated molecular mass of the predicted 402-amino-acid ABCD4 polypeptide was approximately 45.3 kDa. In contrast, Western blot analysis of sheep liver proteins detected a major immunoreactive band with an apparent molecular mass of approximately 37 kDa (Figure 4C).

**Figure 4:**
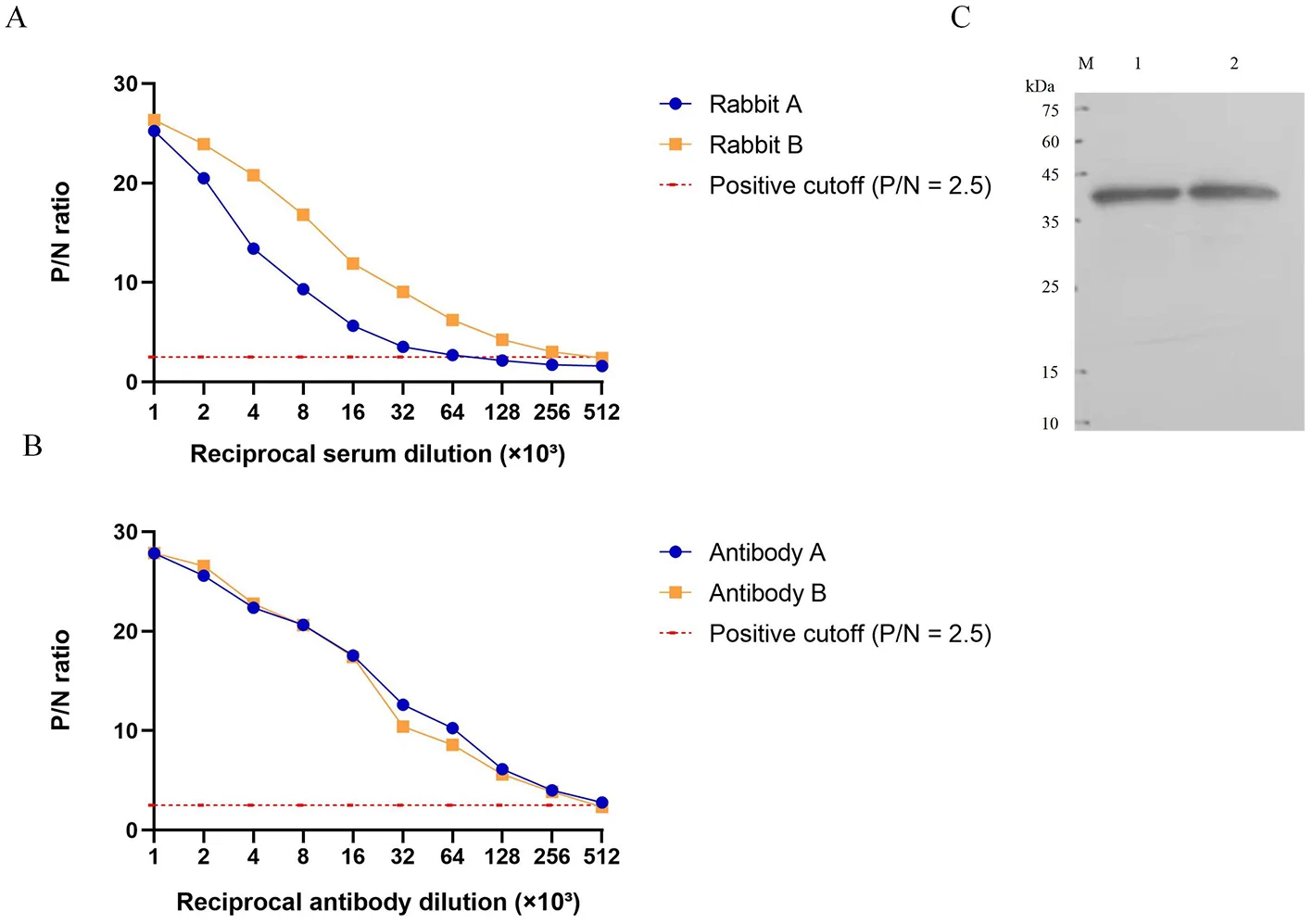
ELISA endpoint-titer determination and immunoreactivity of anti-ABCD4 polyclonal antibodies. **(A)** ELISA titration curves of antisera collected after final bleeding from rabbits A and B. A positive reaction was defined as a P/N ratio ≥2.5, where P/N represents the OD450 value of the sample divided by that of the corresponding negative control. The endpoint titers were 1:64,000 and 1:256,000 for rabbits A and B, respectively. **(B)** ELISA titration curves of the final antibody preparations. Antibody A remained positive at the highest dilution tested (1:512,000), corresponding to an endpoint titer of ≥1:512,000, whereas the endpoint titer of antibody B was 1:256,000. **(C)** Western blot analysis of sheep liver proteins using anti-ABCD4 polyclonal antibody A (lane 1) and antibody B (lane 2). A major immunoreactive band was detected at approximately 37 kDa. M, protein molecular weight marker (kDa).

## 4 Discussion

Although *ABCD4* has been well characterized in humans and mice, its molecular and structural features in economically important animals such as sheep lack in-depth exploration. In this study, a RACE-derived partial *ABCD4* cDNA sequence from Ujimqin sheep was obtained and characterized. Additionally, its prokaryotic recombinant expression, purification, and polyclonal antibody preparation provided reliable tools for future investigations into the biological function of ovine *ABCD4*.

Previous genome-wide association and population-genetic studies have identified *ABCD4* as a candidate gene within genomic regions associated with rib and vertebral-number variation in livestock, including pigs and Ujimqin sheep (22–26). However, *ABCD4* is primarily characterized as a membrane transporter involved in intracellular cobalamin trafficking, and the mechanistic relationship between this transport function and skeletal or vertebral development remains unclear. Therefore, these genetic associations should be interpreted as evidence supporting *ABCD4* as a candidate locus rather than as direct proof that *ABCD4* regulates vertebral-number variation. The present study did not directly investigate skeletal phenotypes or establish a causal relationship between *ABCD4* and vertebral development. Instead, the cDNA sequence, recombinant protein, and polyclonal antisera generated here provide molecular and immunological resources for future studies of *ABCD4* expression, localization, and potential biological roles in tissues relevant to growth and skeletal development.

Although liver tissue was selected as the RNA source because available expression information indicated relatively high *ABCD4* expression in this tissue, liver is not directly representative of vertebral or skeletal development. Therefore, the present study cannot determine the expression pattern or functional role of *ABCD4* in skeletal-development-related tissues. Future studies examining *ABCD4* expression, localization, and function in vertebral, cartilage, and other relevant tissues will be necessary to clarify its potential relationship with vertebral-number variation.

Canonical ABCD4 is an ABC half-transporter containing a six-helix TMD and one NBD per protomer. In contrast, the 402-aa sequence characterized here is N-terminally truncated and does not contain the complete canonical transmembrane architecture. The retained C-terminal nucleotide-binding region therefore supports its ABCD4 origin but does not demonstrate intact transporter function.

In the present study, sequence comparison showed that the recovered region retained conserved ABC-transporter-related features across mammalian ABCD4 sequences. However, because the RACE-derived sequence lacks the N-terminal coding region present in canonical ovine ABCD4 isoforms, these findings should be interpreted as sequence conservation within the recovered region rather than evidence of complete transcript, structural, or functional conservation. The longest RACE-derived cDNA obtained was 1,874 bp and encoded a predicted 402-amino-acid polypeptide corresponding to residues 205–606 of the annotated ovine ABCD4 RefSeq isoform X1.

Prokaryotic expression systems are widely employed for recombinant protein production in a variety of organisms owing to their cost-effectiveness, high yield, and operational simplicity (27). In this study, the recombinant expression conditions for sheep ABCD4 were optimized, and the highest expression level was obtained after induction with 0.5 mM IPTG at 37 °C for 4 h. SDS-PAGE analysis of the purified recombinant protein showed a prominent band with an apparent molecular mass of approximately 37 kDa. Therefore, the approximately 37-kDa band was interpreted conservatively as the experimentally observed apparent molecular mass rather than as confirmation of the predicted molecular size.

The calculated molecular mass of the predicted 402-amino-acid ABCD4 polypeptide was approximately 45.3 kDa. Based on the expression construct, the addition of the N-terminal 6 × His tag resulted in a calculated molecular mass of approximately 46 kDa for the recombinant protein. In contrast, SDS-PAGE and anti-His Western blotting consistently showed an apparent molecular mass of approximately 37 kDa. The basis for this discrepancy remains unclear and may involve anomalous electrophoretic migration, proteolytic degradation, or truncation during recombinant expression. Therefore, the approximately 37-kDa band should be interpreted as the apparent molecular mass of the recombinant product rather than its expected molecular mass. Similarly, the approximately 37–40-kDa immunoreactive band detected in sheep liver cannot be conclusively assigned to endogenous ABCD4 solely on the basis of the present Western blot analysis, particularly because annotated canonical ovine ABCD4 proteins are substantially larger. Further validation, such as peptide competition, genetic depletion, or mass-spectrometric identification, would be required to establish the identity of this band.

The titer of the rabbit anti-ABCD4 polyclonal antibody increased markedly after the third immunization and peaked after the fourth immunization, indicating that the recombinant protein possessed good immunogenicity. Compared with antisera raised against other ABCD family proteins, including ABCD1 and ABCD2, the antiserum generated in this study exhibited specificity and an immune response. These properties support its potential utility in the detection of *ABCD4* expression in sheep tissues, as well as in subcellular localization and functional studies.The generated antiserum showed strong immunoreactivity against the recombinant antigen. However, the present validation is insufficient to establish its specificity for endogenous ovine ABCD4. In particular, the approximately 37–40-kDa immunoreactive band detected in sheep liver cannot be conclusively assigned to endogenous ABCD4 based on the present Western blot analysis alone. Additional orthogonal validation, such as peptide competition, genetic depletion, or mass-spectrometric identification, will be required before the antibody can be reliably applied to endogenous ABCD4 detection, subcellular localization, or functional studies.

## 5 Conclusions

In this study, an ovine *ABCD4* cDNA sequence was characterized from Ujimqin sheep using RACE. A recombinant expression and purification system for ovine *ABCD4* was established in Escherichia coli, and rabbit polyclonal antisera against the recombinant antigen were generated. These molecular and immunological resources provide a foundation for future studies of ABCD4 expression, localization, and biological function in sheep.

## Data Availability

The original contributions presented in the study are included in the article/Supplementary material, further inquiries can be directed to the corresponding authors.
