# Supplementary material for: Cloning and molecular characterization of an ovine ABCD4 cDNA sequence, recombinant protein expression, and polyclonal antibody generation in Ujimqin sheep
Source: Front Vet Sci. 2026 Oct 28;13:1869570. doi: 10.3389/fvets.2026.1869570 (PMC13751471; doi:10.3389/fvets.2026.1869570)
Supplement: Supplementary file 1 [file Data_Sheet_1.docx]

***Supplementary Material***

**1 Supplementary Figures and Tables**

**1.1 Supplementary Figures**

**(A)**

**(B)**

**Supplementary Figure S1.** Multiple sequence alignment of the three 3′ RACE products obtained from Ujimqin sheep with the annotated ovine ABCD4 reference sequence. (A) Enlarged 5′-to-middle portion of the alignment. (B) Enlarged middle-to-3′ portion, including the region in which product 3 diverges from products 1 and 2. For consistency with the revised manuscript, the software-generated labels “Transcript 1–3” correspond to 3′ RACE products 1–3, respectively. The alignment was divided into two enlarged panels solely to improve readability; no sequence information was removed or altered.

**1.2 Supplementary Tables**

| Sequence | mRNA accession | Protein accession | CDS length | Predicted protein length |
| --- | --- | --- | --- | --- |
| Isoform X1 | XM_004010785.4 | XP_004010834.2 | 1,821 bp | 606 aa |
| Isoform X2 | XM_012182043.2 | XP_012037433.1 | 1,791 bp | 596 aa |
| Isoform X3 | XM_012182044.2 | XP_012037434.1 | 1,770 bp | 589 aa |
| Isoform X4 | XM_012182046.2 | XP_012037436.1 | 1,551 bp | 516 aa |
| RACE-derived product 1  (longest sequence) | — | — | 1,209 bp | 402 aa |

**Supplementary Table S1.** Comparison of the RACE-derived ovine ABCD4 cDNA sequence with annotated Ovis aries ABCD4 transcript variants in NCBI.
